# Pain, Pathophysiological Mechanisms, and New Therapeutic Options for Alternative Analgesic Agents in Sheep: A Review and Investigation

**DOI:** 10.3390/ani11030909

**Published:** 2021-03-22

**Authors:** Bogdan Feliks Kania, Danuta Wrońska, Urszula Bracha

**Affiliations:** 1 University Center of Veterinary Medicine Jagiellonian University-Agricultural University, Hugon Kołłątaj Agricultural University in Krakow, Al. Mickiewicza 24/28, 30-059 Krakow, Poland; 2 Department of Animal Physiology and Endocrinology, Animal Breeding and Biology Faculty, Hugon Kołłątaj Agricultural University in Krakow, Al. Mickiewicza 24/28, 30-059 Krakow, Poland; rzwronsk@cyf-kr.edu.pl; 3 Center of Experimental and Innovative Medicine, Hugon Kołłątaj Agricultural University in Krakow, ul. Rędzina 1C, 30-248 Krakow, Poland; urszula.bracha@urk.edu.pl

**Keywords:** pain, pathomechanisms, nonspecific therapy, sheep

## Abstract

**Simple Summary:**

The purpose of this review is to provide current data on the definition and types of pain, describe its neuropharmacological and pathological mechanisms, and present a comparative analysis of the results obtained after intracerebroventricular (ICV) infusion of voltage-gated calcium channel inhibitors (VGCCIs) such as diltiazem, nifedipine, and verapamil, cholecystokinin receptor antagonists (PD, proglumide), and glutamatergic receptor antagonists (L-AP_3_, DL-AP_3_) during experimental distension of the duodenal and/or descending colonic wall. This method was used as a model for suppressing pain in sheep based on viscero-visceral inhibitory reflex, measured by the inhibition of behavioral symptoms of stress, degree of reticulo-ruminal motility, and changes in plasma cortisol and catecholamine concentration. After ICV infusion, all tested substances suppressed, to varying degrees, the viscero-visceral inhibitory reflex, tachycardia, hyperventilation, bleating, and gnashing of the teeth, whereas they increased the levels of cortisol and plasma catecholamines in sheep. These substances could be potential non-narcotic agents for the treatment of visceral intestinal pain (intestinal colic) in sheep, but clinical confirmation of the substances’ efficacy for treating intestinal colic is needed.

**Abstract:**

Relief from suffering is the guiding principle of medical and veterinary ethics. Medical care for animals should be carried out to meet all welfare conditions. The need for pain management is demonstrated by recent monographs devoting attention to this urgent ethical need. Little data, however, are available on the prevention and attenuation of pain in sheep. After administration of narcotic analgesics used for severe visceral pain, sheep react with a state of excitement. Therefore, it was decided to experimentally investigate the usefulness of potential non-narcotic drugs to relieve pain in sheep with intestinal colic caused by 10 min of mechanical distension of their duodenal and/or descending colonic wall. The results indicate the potential usefulness of VGCCIs (diltiazem, nifedipine, verapamil), cholecystokinin receptor antagonists (PD, proglumide), and metabotropic glutaminergic receptor antagonists (mGluRAs), such as L-AP_3_, DL-AP_3_. As a premedication, these substances prevented the occurrence of symptoms of acute intestinal pain including atony of reticulo-rumen, tachycardia, hyperventilation, moaning, gnashing of teeth, hypercortisolemia, and catecholaminemia; hence, these substances are considered potential agents in the treatment of sheep visceral pain.

## 1. Introduction

Since the earliest times, living organisms receive stimuli from their environment. Many of these stimuli are beneficial; however, some stimuli cause discomfort to individuals. These adverse stimuli usually cause discomfort of a painful (nociceptive), even damaging (*noxa*), and sometimes toxic nature. Organisms have to avoid or counter situations when the body’s homeostasis is disturbed by these adverse factors or painful stimuli [1], regardless of the nature of the stimulus (physical, chemical, or psychological) [2]. Homeostasis is restored in humans by neuroendocrine reactions or, when these are not sufficient, e.g., when the nociceptive factor exceeds the adaptability of organisms, by using substances that restore a sense of comfort. The same procedure applies to all vertebrate animals that react similarly to humans.

## 2. Historical Outline

Pain has been known since the dawn of time, for as long as living organisms have existed. Considered as a complex physiological phenomenon, pain provides evidence of the effective impact of the external and internal environment on organisms. Pain is regarded as a special type of experience that results in various affective reactions with strong emotional components. As far back as 1550 BCE, opium was used to relieve pain and was described on Ebers papyrus. Hippocrates (460–377 BC), named by posterity the father of medicine, claimed that pain is one of the actual symptoms of disease. According to him, the brain was the main location of pain sensation; therefore, it is not surprising that, for many centuries, pain was considered to be a symptom of disease of the soul and heart. Galen (130–201) and Avicenna (980–1037) were pioneers in identifying the location and causes of pain formation, claiming that the phenomenon of pain develops in the brain and nerves because of sudden shifts in the distribution of body fluids and changes in tissue continuity. Leonardo da Vinci (1442–1519) presented a still valid hypothesis that pain was associated with touch sensation, being a consequence of an excessive stimulus action, currently termed a noxious stimulus. Descartes (1569–1650) presented in his work “*Tractatus de homine*”, a description of a hypothetical path through which impulses pass from the site of action to a center in the brain. It was not until the 20th century that a breakthrough in research occurred concerning on pain perception. The results of experimental studies carried out in the last two decades have provided biological evidence for progress in the recognition of both the morphological and the functional basis of the phenomenon of pain perception. However, controversies and inconsistencies of this fascinating phenomenon are still evident. In particular, in recent years, other endogenous opioids, termed endogenous morphines (endomorphin-1, endomorphin-2), were discovered [3,4,5] and the presence and structure of a presumed endogenous pain transmitter (nociceptin) was reported, but the transmitter’s biological role in living organisms is still being elucidated [6,7].

Since the time of Sertürner, who, in 1804, isolated the substance he named after the god of sleep, Morpheus, it is known that the compound that reduces or abolishes the adverse effects of pain stimuli is morphine, a phenanthrene opium alkaloid from *Papaver somniferum* with analgesic and narcotic effects (addictive and tolerance-inducing). Fifty years after its isolation, morphine was added to the arsenal of drugs used in the treatment of postoperative and chronic pain [8].

**Figure 1:**
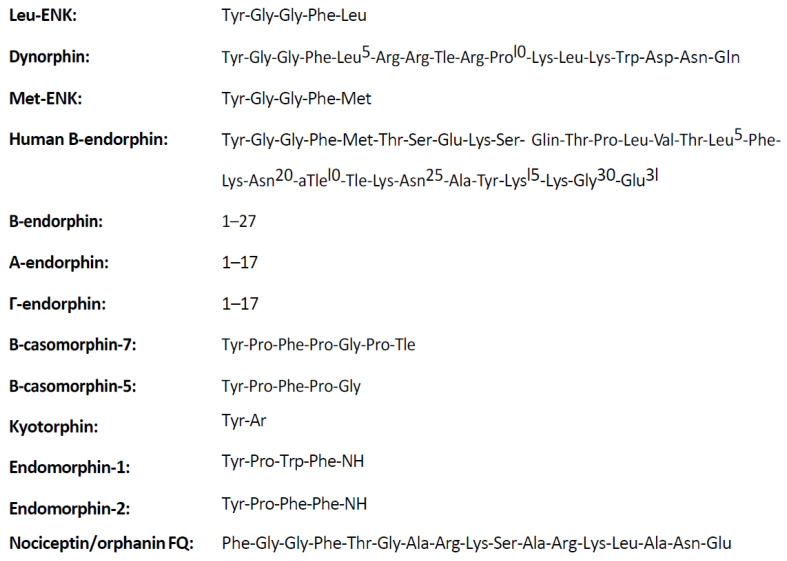
Structure of endogenous opioid peptides (EOPs) [1].

Alleviation of endogenous pain by an exogenous alkaloid brought up an assumption that a morphine-specific locus of action must exist in living organisms. Over time, the presence of receptors for morphine, later named opioid receptors, was validated, and other receptors were subsequently identified, named, and localized. The existence of three basic groups of opioid receptors (µ, δ, and κ) was determined, and the division into subtypes of these groups (µ_1_, µ_2_, δ_1_, δ_2_, κ_1_, κ_2_, and κ_3_) was suggested by some authors (Table 1). These receptors are distributed over the central and peripheral nervous system and organs (Table 2) and are present at the highest density in the structures responsible for reception and conductivity of pain stimuli in humans and other vertebrates [3,4,7]. Furthermore, the presence of various opioid receptors in the organism’s structures suggested the existence of endogenous substances specific for these receptors. The existence of compounds with morphine-like activity was, thus, proven and named endogenous morphine (endorphins), which are peptides with opioid activity (endogenous opioid peptides; EOPs) (Figure 1). To distinguish endorphins from opioid-like substances of exogenous origin, exogenous substances were named opiates. Subsequently, numerous endorphins were identified in the body (Figure 1) [4,6].

In the wake of the achievements of theoretical sciences, exogenous substances that mimic the effects of endogenous endorphins were soon developed. Some of the obtained opiate substances proved to be 1000–80,000 times more potent in analgesic tests than their protoplast, morphine [1,10]. However, these substances have certain adverse effects in some species, particularly ruminants [1,11]. Effects are excitant and sympathomimetic, and, in humans, there is stimulation of the reward system (addiction) and inducement of tolerance to these drugs. Differences are due to the fact that companion animals have well-developed analgesic and preanesthetic processes [8,11] and farm animals suffer from a constant deficiency of these processes [12,13].

Non-narcotic analgesics for suitability for use in ruminant anesthesiology (α_2_-adrenergic receptor agonists, nonsteroidal anti-inflammatory drugs (NSAIDs) [8], local anesthetics, ionotropic glutaminergic receptor antagonists (iGluRAs) such as ketamine, and voltage-gated calcium channel inhibitors (VGCCIs; diltiazem, nifedipine, verapamil, type 1 cholecsystokinin receptor antagonists—CCK_1_)) have been the subject of recent research interests. Sheep, for example, have many behavioral and biochemical reactions that are similar to human reactions [13]. This was a main reason for undertaking this study to investigate a new method for determining visceral pain during the viscero-visceral inhibitory reflex test in use since the 1990s [14].

## 3. Definitions of Pain

The phenomenon of pain sensation is defined differently, depending on the specialty. From the point of view of psychology or psychiatry, pain is a difficult to define and a subjective experience. Being a result of awareness of nerve impulses reaching the brain and caused by noxious stimuli of adequate strength, pain can be also defined as a psychic factor, triggering defensive reflexes. From a biological point of view, pain is a warning signal about a danger or tissue injury. Pain is a sensory impression, formed by the action of various stimuli that damage tissues; therefore, pain provides information about the action site of the harmful factor (stressor). Pain can be experienced as a local sensation or as widespread pain when the response to stimulus is intense and the effect of the damage lasts for an extended duration. Controversies still exist regarding nomenclature and definitions specifying the phenomenon of pain. According to the Taxonomy Committee of the International Association for the Study of Pain (IASP), pain is defined as an unpleasant sensory and emotional experience associated with, or resembling that associated with, actual or potential tissue damage [15].

Following this definition, pain is a psychological, subjective, and emotional phenomenon, related not only to the stimulus causing it, but also to the memory of previous experiences [14]. This phenomenon especially applies to humans, but does this phenomenon also concern animals? For example, in rat species, viscero-visceral inhibitory reflex occurs and is exemplified by triple, strong, weekly, 5 min long dilatation of the colon that cause inhibition of gastric motility and intense pain. Researchers are trying to interpret this phenomenon as a viscero-visceral inhibitory reflex [12,13,16,17,18]. If the rat reacts with identical inhibition of gastric motility after placing an empty balloon in the colon ten days after the previous dilatation, it should be assumed that these phenomena occur as a reflex (presumably memorized). In such case, there would be no painful, mechanical dilatation of the colon, nor would any behavioral symptoms or lesions be evident that would indicate irritation of the colonic mucosa by the inserted balloon, such as defecation, urination, hyperventilation, tachycardia, squealing, intensification of locomotor activity, or increased release of stress hormones (catecholamines, cortisol, adrenocorticotropic hormone (ACTH), vasopressin, aldosterone, and others) [18].

## 4. Types of Pain

According to nociceptive stimuli action areas, the following types of pain are distinguished: (1) superficial (skin) pain, (2) deep pain resulting from damage of the musculoskeletal system, and (3) visceral pain, a consequence of ongoing disease processes in internal organs [19]. The first two types are called physiological pain. Superficial pain is experienced because of the action of nociceptive stimuli on the pain receptors in the skin (nociceptors), whereas deep pain can cause bone injury, damage, or straining of the articular capsule or tendon. For each type of pain, as mentioned above, the sensations may be local or diffuse pain. Pain can also be divided into physiological and pathological types. Pathological pain includes postoperative, chronic, inflammatory, and cancer pains, as well as pain associated with muscle ischemia (ischemic pain), rheumatoid pain (related to musculoskeletal disorders), and neuropathic pain that occurs in nerve diseases, whereas physiological pain is derived from nondisease stimuli [20,21].

## 5. Neuropharmacological Basics of Pain

Factors that cause the release of tissue mediators of pain, such as substance P (SP), bradykinin (BK), histamine, acetylcholine (Ach), serotonin or 5-hydroxytryptamine (5-HT), and prostaglandins (PG), are termed kallikrein-type sensory peptides. Peroxides from PG exhibit nociceptive effects in animal tissues. These compounds, by stimulating Cdr and/or Aδ type nociceptive fibers, increase the release of SP and calcitonin gene-related peptide (CGRP), which in turn excite neurons that conduct nociceptive stimuli and enhance the intensity of pain perception. As mentioned previously, the modulation of pain stimulus transmission in living organisms is achieved by “gating” the entry of nociceptive stimuli by their own endogenous morphine, EOP, including endorphins, enkephalinergic (ENK) neurons, and dynorphins, at the level of the spinal cord (first gate), thalamus (second gate,) and 5-HT- and noradrenergic (NE-ergic) through nonopioidergic pathways. Thus, the intensity of stimuli causing nociception, reaching the cortex, and experienced as pain is the result of excitations of the ascending and descending systems [3,4,20].

### Consequences of Nociceptive Sensations and Their Therapeutic Implications

Some claim that animals do not experience anxiety in the psychological sense, that is, mental states that cause pain of an unspecified etiology. According to others, animals react only with fear, like that felt by a human before a visit to the dentist. If this is the case, how does one explain a dog whining, squeaking, barking, biting the door, jumping, or howling at the sight of the departing owner when left alone? This and many other questions arise, especially in the aspect of clinical symptoms of various types of pain. Depending on its intensity and duration, the symptoms of pain in animals are behavioral and biochemical changes, such as moaning, roaring, bleating, defecation and/or urination, atrophic changes of the adrenal glands, release of stress hormones into the blood, increased susceptibility to infections, reduced growth, reproductive disorders, and many more.

Strong analgesics (narcotics) in veterinary medicine are used as a premedication and, to a lesser extent, during the postoperative period (in which NSAIDs are mostly utilized and have no effect on neoplastic processes) [1,12]. The domain of the use of analgesics (in the strict sense) in combating visceral, post-traumatic (acute), chronic, and cancer pain is neglected [1,10]. The adverse consequence of omitting antinociceptive therapy in animals is increased stress, especially increased symptoms associated with an alarm reaction (increased levels of ACTH, E, NE, cortisol, vasopressin, renin, angiotensin II, aldosterone, and glucose and decreased levels of insulin and testosterone in blood). Known symptoms of the excessive pain, both acute and chronic, constitute the indication and necessity to use analgesics, because those are substances that can reduce suffering. The therapeutic utility of various analgesic drugs in different types of nociception in animals is discussed below.

## 6. Pain as a Stress Factor

Pain, regardless of its trigger, causes the same symptoms as stress [1,22]. What is more surprising, the same or similar structures of the central nervous system and their mediators are the cause of the different behavior of animals and the somatic changes in their organs due to the influence of the nociceptive factor. The biological basis and consequences of pain in small animals have been sufficiently presented in numerous publications [8,23,24]. Their conclusion is that treating pain in animals is just as important as in humans. This conclusion applies to postoperative, traumatic pain, which is acute and during which animals also experience fear, anxiety, or confusion [25], as well as chronic, blunt pain, often with diffuse projection, resulting from osteoarthritis, cancer, chest surgery or implantation of an artificial joint [24]. The veterinarian’s primary task, therefore, in cooperation with the owner, is to relieve or eliminate pain to improve the quality of life of individuals, especially those who are elderly, in poor condition, or undergoing prolonged chemo- or radiotherapy [26].

Relief of suffering is the basic rule of medical ethics, including veterinary medicine. Animals need humane treatment based on the modern achievements of veterinary neuropharmacology. High-intensity pain requires the use of a narcotic analgesic, which should not be used during the manifestation of symptoms of patients’ increased suffering, but in advance, to avoid dilemmas concerning which drugs to use such as analgesics, anxiolytics, sedatives, or muscle relaxants [10]. Medical care for the animal should be carried out in such a manner, to meet all the conditions of its welfare [27]. The necessity of pain management is also confirmed by recently published monographs devoted to this urgent ethical need [22,28,29].

## 7. Pain Conduction Pathways

### 7.1. Perception of Stimuli and Nociceptive Pathways

Receiving stimuli from the environment through the skin is possible due to receptors of touch, temperature, and pain [30]. Skin receptors, especially of touch, are found in miniature, encapsulated receiving bodies called Pacinian corpuscles, Merkel cells, and Meissner’s corpuscles. Other skin receptors are free endings of nerve fibers [29]. Cold sensory receptors are in the surface layer of the skin, bordering the epidermis. Receptors located in the deeper layers of the skin are heat sensory receptors. Impulses of superficial sensation are sent via thick myelinated Aβ and Aδ fibers and thin autonomic unmyelinated Cdr fibers [19,21]. The receptors of the pain sensation in the skin are nociceptors. Nociceptors are the free ends of thin myelinated fibers of the Aδ subtype (2 to 5 mm diameter), conducting impulses at a speed of 12–30 m/s, and unmyelinated Cdr type fibers (0.4–1.2 mm diameter), conducting impulses at a speed of 0.5–2 m/s [31], explaining why pain is felt in biphasic manner. In the first stage, pain is acute and, after some time, often becomes dull, diffuse, and burning pain. During excitation of sensory nerves of the human skin, the induction of activity in the Aδ subtype fibers causes the feeling of acute, well-localized pain, and the stimulation of Cdr class fibers elicits the sensation of dull, burning pain [24,25].

The ability to receive pain stimuli is called nociception. Nociception is predominantly accompanied by algesia, the sensation of pain [21]. An animal’s ability to feel pain is assessed on the basis of nociceptive reflexes utilizing the hot plate method, tail flick test, or writhing syndrome. Nociceptive sensations are not just a consequence of touch receptors excitation. Algesia can be caused by stimulation of the eyes’ photoreceptors or hearing receptors if the intensity of the light or sound significantly exceeds the normal values typical for a specific sensory modality [31]. Therefore, it can be concluded that nociceptors, unlike other types of receptors (e.g., mechano-, presso-, and thermo-electromagnetic receptors), do not receive specific stimuli. Nociceptor excitation is caused by strong, tissue-damaging mechanical stimuli, as well as chemical, electrical, or thermal stimuli [27]. Class Aδ fiber nociceptors react to mechanical stimuli, whereas some respond to a higher temperature or to the skin cooling [32]. The ends of Cdr fibers are sensitive to thermal stimuli in the temperature range from 41 to 49 °C [29]. These nociceptors are also excited by mechanical stimuli (pressure, pricking), but their sensitivity is lower than that of the Aδ subtype fibers [29,30]. The ends of Cdr fibers are also sensitive to chemicals released from damaged tissue and stimuli of other categories; therefore, they were named polymodal receptors [29].

Under physiological conditions, nociception is associated with stimulation of electrical activity of thin sensory afferent fibers located in the peripheral nerves. These fibers have sensory endings in peripheral tissues and can be excited by stimuli of different nature (mechanical, chemical, thermal, biological) [33]. Unlike other types of mechanical or temperature receptors, these fibers require high-intensity stimuli to be stimulated. Under physiological conditions, excitation of these fibers is induced only by noxious stimuli, capable of causing various degrees of tissue injury. Measurement of the activity of a single nerve fiber in human proved that a stimulus sufficient to stimulate these thin afferent fibers also causes pain sensation [30]. In the damaged tissue, the proteolytic enzymes, tissue kallikreins, are activated. They detach active polypeptides, kinins, from active tissue proteins, termed kininogens. Subsequently, kinins depolarize nociceptors and trigger series of nociceptive impulses in primary afferent sensory fibers. In addition to kinins, other “sensory peptides” are released in damaged tissues.

### 7.2. Afferent Conduction Pathways of Nociception

**Figure 2:**
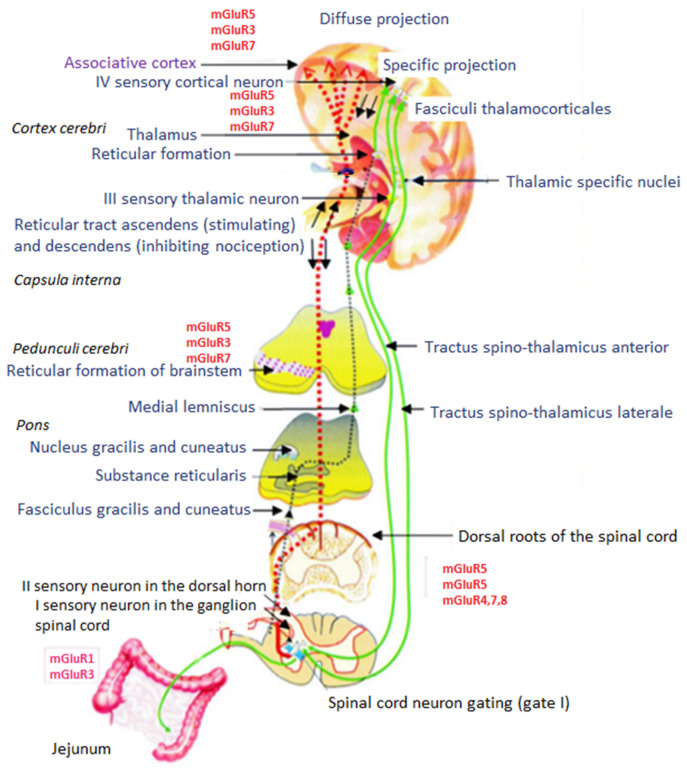
Diagram of specific ascending pathways conducting nociceptive (pain) stimuli and nonspecific ascending (excitatory) and descending (antinociceptive) pathways of the reticular formation. The figure shows pathways of pain conduction from nociceptors located in the intestine, through class IV sensory neurons, to sensory areas of the cerebral cortex and “gating” systems, inhibiting the transmission of nociceptive stimuli in the spinal cord (first gate) and thalamus (second gate). A detailed description of metabotropic glutaminergic receptor (mGluR)-mediated transmission and inhibition of nociception can be found in the text (according to [34]).

Nociceptors are free nerve endings specifically adapted to receive nociceptive stimuli. After their depolarization by a damaging factor, excitation is conducted by primary afferent myelinated Cdr fibers or myelinated fibers of the Aδ subtype with mediator SP and ACh, which is a chemical compound that causes pain. As axons of bipolar neurons located in the spinal ganglia, the sensory fibers merge into sensory nerves and then enter the spinal cord through the dorsal roots (Figure 2). These bodies of bipolar neurons form the abovementioned spinal ganglia, constituting the first sensory neuron that conducts nociceptive stimuli (Figure 2). Neuropeptides, also termed “sensory neuropeptides”, are released from the ends of the primary axons of nociceptive fibers (SP-ergic) that secrete pain substances to the synapse they form with interneurons of substantia gelatinosa (SP, CGRP, vasoactive intestinal peptide (VIP), somatostatin (SRIF), and/or ACh, galanin). SP, VIP, and CGRP have an excitation effect on the second sensory neuron in the dorsal horns of the spinal cord. Galanin has an inhibitory effect, while somatostatin has both an inhibitory and a stimulating effect [1].

The abovementioned peptides are also released from the ends of the primary nociceptive fibers in tissues such as skin, joints, internal organs, or skeletal muscles. These “sensory neuropeptides” dilate blood vessels and increase their permeability, thereby causing swelling, while they also act in a nociceptive fashion. Moreover, these peptides enhance the division of mast cells, fibroblasts, and macrophages in tissues. Presumably, SP facilitates the release of from mastocytes [27].

The excitation from interneurons, depolarized by neuropeptides because of the action of a nociceptive factor, is transmitted through other intermediate neurons (second sensory neuron) (Figure 2) to motoneurons of the anterior horns and to neurons located in substantia gelatinosa and nucleus proprius of the anterior horns. Ganglion cell axons (primary nociceptive fiber endings), located in the spinal canal, form synapses with enkephalinergic (ENK) interneurons of substantia gelatinosa that inhibit the transmission of nociceptive stimuli. Excitation of ENK neurons, for example, by SP, enhances the release of ENK, which suppresses nociceptive stimulation by reducing SP release from the ends of the primary nociceptive fibers, known as the first “gating” neuron of pain input. The transmission of the excitation state to motoneurons is explained by the withdrawal reflex, often used in pharmacological studies. The neuron bodies of substantia gelatinosa and nucleus proprius receive excitation from the ends of primary nociceptive fibers. Their axons pass to the other side of the spinal cord to the lateral part of the white matter and, after this intersection, they head upward, giving an ascending pain projection to the thalamus. The axons continue along the spinal cord and brainstem before reaching the thalamus, forming lateral and medial spinothalamic tracts (Figure 2). Through these tracts, nociceptive stimuli are conducted mainly to the ventral posterior nuclei. Some fibers also project into the midline and intralaminar thalamic nuclei. In the thalamus, the intensity of the nociceptive stimulus is assessed [21,30]. From the spinothalamic tracts, primarily the medial ones, collaterals branch out to neurons of the reticular formation (*formatio reticularis*). Therefore, as a consequence of strong nociception, the autonomic nervous system is stimulated and symptoms such as mydriasis, hyperhidrosis (*sudorrhoea*), tachycardia, respiratory system stimulation, and sometimes even shock can be observed. The fourth neuron of afferent nociceptive pathways begins predominantly in the ventral posterolateral thalamic nuclei, and its projections reach the cerebral cortex directly (specific pathways) or indirectly (diffuse projection) through the nonspecific pathways of the reticular formation of the brain stem. Perception of the phenomenon of nociception occurs in the cerebral cortex.

Studies on functional brain imaging have identified brain areas involved in pain sensation. These are sensory, discriminatory (inhibitory) regions, such as the primary and secondary somatosensory cortex, thalamus, and posterior part of the insula, as well as affective cognitive areas of the anterior part of the insula, prefrontal cortex, and part of the cingulate cortex [35]. Raver et al. [36] recently determined the existence of a pathway leading from the amygdala to the parabrachial nucleus that modulates both pain sensation and chronic pain.

### 7.3. Efferent Inhibitory Pathways of Nociception

**Figure 3:**
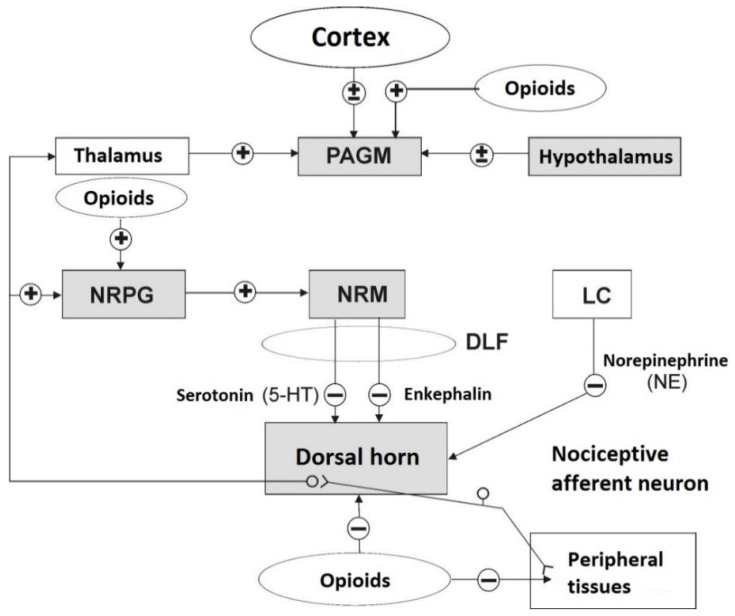
Diagram of the descending control system with the main sites of opioid action on pain transmission. Opioids stimulate neurons in the periaqueductal gray matter (PAGM) and in the nucleus reticularis paragigantocellularis (NRPG), which in turn projects into the antero-ventral region of the medulla oblongata, where the nucleus raphe magnus (NRM) is located. From NRM, neurons containing 5-hydroxytryptamine (5-HT) and enkephalinergic (ENK) neurons head to substantia gelatinosa of the spinal dorsal horn, where they inhibit the transmission of nociceptive impulses. Opioids also have a direct inhibitory effect on the spinal dorsal horn, as well as on the peripheral endings (nociceptors) of the nociceptive afferent neurons. Locus coeruleus (LC) directs noradrenergic (NE-ergic) neurons to the dorsal horn, in which they also inhibit the transfer of nociceptive stimuli. The pathways depicted in this diagram constitute a significant simplification; however, it reflects the overall organization of supraspinal control mechanisms enabling pain inhibition. Glossary: DLF—dorsolateral funiculus; + stimulation; − inhibition; shaded fields represent regions (structures) with a high concentration of opioid peptides (based on [37]).

Descending pathways that inhibit the transfer of pain impulses to the cerebral cortex begin in the middle part of the midbrain, known as the periaqueductal gray matter (PAGM) (Figure 3). ENK-ergic fibers emerge from PAGM with an inhibitory synapse on the serotonergic neurons of the nucleus raphe magnus (NRM) (second “gate”). The axons of these neurons form synapses with the primary SP-ergic nociceptive fibers of the substantia gelatinosa of the spinal cord. Irrespective of the 5-HT-ergic inhibition of pain stimuli transmission, other non-opioidergic pathways that inhibit nociceptive transmission exist, starting in neurons of the nucleus reticularis paragigantocellularis (NRPG). The axons of these neurons are NE-ergic and terminate with synapses on the bodies of the interneurons of the substantia gelatinosa of the spinal cord. Norepinephrine, released from these axons, as a reaction to nociceptive stimulus, excites ENK-ergic interneurons. This reaction causes the increase release of ENK which inhibits the release of SP from the primary ends of the SP-ergic nociceptive fibers, i.e., inhibits the stimulation of the neuron transmitting nociceptive stimuli. Therefore, it can be concluded that there is an in vivo arrangement of the descending system regulating the spread of nociceptive stimuli of opioid (ENK) and non-opioid (5-HT- and NE-ergic) nature, modulating the transmission and inhibiting the ability to feel pain in living creatures (Figure 3).

## 8. Opioid Receptors

There are four major subtypes of opioid receptors. The opioid growth factor receptor (OGFr) was originally discovered and named as a new opioid receptor zeta [38]. However, it was subsequently found that it shares little sequence similarity with the other opioid receptors and has a different function.

In 1954, a hypothesis was made about the existence of a central receptor stimulated by morphine and other exogenous opioids. This theory was confirmed by Goldstein in 1971, who used 3H- and 14C-levorphanol specifically bound by synaptosomes of brain homogenate [9,39]. In 1975, research teams led by Simon [40], Snyder [41], Terenius [42], and Martin in 1976 [9] confirmed the presence of three types of receptors (μ, δ, κ) whose ligands are opiates, now called exogenous opioids, in the nervous system of mice and rats. Identification of opioid receptors, suppression of post-stress analgesia by naloxone (stereospecific opioid antagonist), and induction of hyperalgesia, sometimes found after the use of naloxone alone, led to the presumption of existence of endogenous substances imitating exogenous opioid activity and binding to the same opioid receptors. A few years later, EOPs with biological activity imitating the effects of morphine (phenentrene alkaloid, an opium component acquired from the juice of the immature poppy seeds of the *Papaver somniferum* plant) were obtained and preliminarily characterized. Cox [28] and Hughes et al. [39] have independently isolated the following EOPs: leucine-ENK (Leu-ENK), methionine-ENK (Met-ENK), and α- and γ-endorphin from the alkaloid that is the protoplast of other narcotic analgesics (Figure 3, Table 3). All opioid peptides are called endorphins and include β-endorphin, ENKs, dynorphin, and casomorphin. In the β-endorphin molecule (β-LPH61-91), chains with the amino-acid sequence of α-endorphin (β-LPH61-76) and γ-endorphin (β-LPH61-77) have been distinguished and are products of β-endorphin degradation [29].

The development of radioreceptor, radio-competitive, and pharmacodynamic methods have enabled the identification of over 20 EOPs containing pentapeptide chains. According to the British researchers [29], it was Hughes and Kosterlitz [39] who were the first in the world to isolate two pentapeptides from the brain in 1973. These pentapeptides showed strong competition with morphine-like agents in binding brain opioid receptors with pharmacological features closely resembling those of morphine. Isolation of opioid receptors and endorphins has been recognized as one of the greatest discoveries in biological research since the detection of the antibacterial properties of penicillin.

## 9. Endogenous Opioid Peptides (EOPs)

EOPs and their receptors are found in the central nervous system (CNS), peripheral nervous system (PNS), intestines, and immune system. EOPs can act as transmitters or modulate synaptic activities of primary transmitters. Data indicate that EOPs are involved in central and peripheral antinociception, motor activity, nutrition, sexual behavior, breathing, and body temperature regulation, as well as cardiovascular and gastrointestinal functions. Released from endocrine organs, EOPs take part in regulating the discharge of various hormones and modulating immune functions. EOPs are involved in the function of the reward system, learning, memorizing, and emotional states. EOP systems play an important role in modulation and adaptation of the body to challenges. During the resting phase, EOPs are released in relatively small amounts; however, during intense excitement, they are discharged in large quantities. EOPs appear to be involved in brain diseases such as pain, addiction, depression, and anxiety disorders. The research progress that has been made in recent years in the field of pharmacology, genomics, and complex genetics opens the possibility of studying the role of EOPs and their receptors in various brain diseases and discovering new directions for research on new opioid therapies.

**Figure 4:**
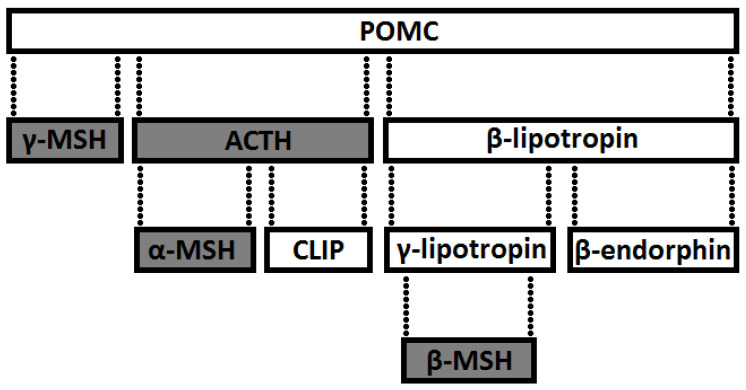
Scheme depicting the structure of biologically active fragments of prohormones as precursors of opioid peptides. Signal peptides are marked as shaded spaces (modified according to [29]).

EOPs, defined as peptides with pharmacological effects like exogenous opioids, are encoded by three different genes whose products are proopiomelanocortin (POMC1-267), preproencephalin (preproENK), and preprodynorphin. Thus, in the human and animal body, their production is similar to hormones or peptide neuromodulators, by separating (cutting off) active fragments from macromolecular precursors (Figure 4). The structure of three protein opioid precursors indicates the location of the opioid and other peptides in the amino-acid sequence of the compound molecule. The peptides located in the chain are linked together by two basic amino acids. These links are the site of the cutting action of enzymes (proteolytic fragmentation).

### 9.1. Classification

Depending on the type of precursor, EOPs are divided into the following:

- derivatives of POMC—precursor of an ACTH-releasing factor (POMC1-39) and β-lipotropin (β-LPH), which is part of the POMC1-91 chain. Endorphins are formed from β-LPH1-91 (β = β-LPH61-91, γ = β-LPH65-77 and endorphin α = βLPH61-76),
- preproencephalin A derivatives (Leu- and Met-ENK),
- hypothalamic preprodynorphine derivatives (dynorphin A and B, l-neo-endorphin, rimorphin),
- exorphins that are also endorphins (milk casomorphin),
- endomorphins (endomorphin-1 and endomorphin-2),
- nociceptin (orphanin).

In the first three families of EOP precursors, each precursor contains an opioid and other peptides. These peptides are bound by adjacent pairs of amino acids, which are also the site of action of the enzyme that cleaves macromolecular peptides during proteolytic fragmentation. Discoverers of ENK [29] found that the molecule of pituitary hormone, β-lipotropin (β-LPH), contains repeated sequences of met-ENK (Figure 1). In the β-LPH molecule, the presence of α-, β-, and γ-endorphin was determined. Enkephalins are derived from products of other genes, i.e., those producing proENKs and prodynorphins. POMC itself is also a source of ACTH, melanocyte-stimulating hormones (MSH), and β-endorphin but is not a source of ENK. The expression of protein precursors changes significantly in various brain tissues and structures. For example, proopiomelanocortin and its derivatives are found mainly in the pituitary and hypothalamus, while ENK and their precursors are located throughout the CNS, PNS, and other organs, including the adrenal medulla [29]. Immunofluorescence studies have shown that these peptides and their precursors are closely associated with their specific cells, and they are associated with the different processes that facilitate the production of various peptides from the same precursors. The peptides can also be found in various tissues and areas of the brain. In the brain, beta-endorphin is located mainly in neurons projecting from the hypothalamus to the thalamus and into the brainstem. ENKs are found primarily in small interneurons in various areas of the brain [29]. Members of the peptide family are independently represented in the genome, but their differentiation can also occur through gene splicing or during post-translational changes of prohormone [29].

### 9.2. Other Opioid Mediators of Nociception

Various metabolites and chemical substances are released from damaged or ischemic cells and inflamed tissues. These include ATP, protons (produced by lactic acid), 5-HT, histamine, and K^+^ ions. Some of these substances interfere with the nociceptive nerve endings functions.

ATP irritates nociceptive nerve endings by acting on homomeric P2X_3_ receptors or heteromeric P2X_2_/P2X_3_ receptors of ligand-gated ion channels that are selectively present on these neurons. Down regulation of P2X_3_ receptor activity means that antisense DNA reduces post-inflammatory pain. Other P2X receptors (P2X_4_ and P2X_7_) present in the microglia and spinal cord, when activated, release cytokines and chemokines that can act on adjacent neurons and facilitate hypersensitivity induction. ATP and other purine mediators, such as adenosine, also play a role in spinal dorsal horns; thus, other types of purine receptors may be a target for new analgesics in the future. On periphery, adenosine acts dually, causing analgesia by affecting A_1_ receptors and acting in a nociceptive manner by stimulating A_2_ receptors.

Low pH stimulates nociceptive afferent neurons by opening proton-activated ion channels (acid-sensitive channels) and by facilitating transient receptor potential cation channel subfamily V member (TRPV1). Although 5-HT stimulates nociceptive neurons, it plays a minor role in nociception [30]. Histamine also stimulates nociceptive neurons but causes irritation rather than pain. Histamine and 5-HT are both released locally in inflammatory processes.

The nociceptive factor causes the release of tissue mediators of pain, such as SP, BK, histamine, ACH, 5-HT, NA, PG, and other substances including glutamate, ATP, protons (from lactic acid), and K^+^ ions [43]. It is currently assumed that it is not PGs, but their peroxides that exhibit nociceptive effects in animal tissues. These compounds, by stimulating Cdr and/or A δ type nociceptive fibers, increase the release of SP and CGRP, which in turn excite neurons that conduct nociceptive stimuli and enhance the intensity of pain perception. As mentioned previously, the modulation of the pain stimulus transmission in living organisms is achieved by “gating” the entry of nociceptive stimuli by their own EOPs such as endorphins, ENKs, and dynorphins, at the level of the spinal cord (first gate) and thalamus (second gate), as well as through non-opioidergic pathways, such as 5-HT- and NE-ergic (Figure 3). Thus, the intensity of stimuli causing nociception, reaching the cortex, and experienced as pain is the result of excitations of the ascending and descending systems inhibiting the transmission of pain-triggering stimuli, acting in an antinociceptive manner.

## 10. Exogenous Opioids (Opiates)

Indication for use of analgesics is as short-term general anesthesia enabling minor procedures such as tartar removal, removal of foreign bodies from the mouth and esophagus, incision of abscess, change of dressings, taking X-rays, or clinical examination of aggressive and excitable animals. Full anesthesia utilizing a combination of opiates with other anesthetics is used to induce general anesthesia, for example, during fracture surgery, sprain reposition, castration, amputation, caesarean section, and laparotomy.

## 11. Visceral/Intestinal Pain

Visceral pain is the most common type of pain encountered in clinical practice. Until recently, it was regarded as a variant of somatic pain, but significant differences between these types or pain have been discovered [44]. Visceral pain is described as an unpleasant sensation from the organs of the chest, abdomen, and pelvis, and it is difficult to locate precisely, often affecting individual sensory fields, known as Head’s zones. Visceral pain is associated with stimulation of the autonomic system, which is manifested by nausea, vomiting, palpitations, sweating, and anxiety [45,46].

The functions of internal organs are regulated by the autonomic nervous system, which consists of centripetal fibers running from intero-receptors, and centrifugal fibers supplying the visceral smooth muscles. Visceral pain receptors are characterized by a significantly lower density of occurrence compared to receptors in the skin; hence, visceral pain is difficult to locate, diffuse, and indistinctly felt [46]. Activation of these receptors is associated with excessive distension or contraction of the organ or the presence of inflammation within the tissues. The cause of intense pain may be intestinal colic, occurring during obstruction of this section of the digestive tract, caused by severe intestinal contraction above the obstruction site [44].

The mechanism of visceral pain formation remains unclear. However, it is known that there are two classes of nociceptive receptors within internal organs. They have a polymodal nature, i.e., they react to various types of stimuli (chemical, mechanical, thermal). Receptors belonging to the first class have a high excitability threshold. They react primarily to mechanical, often noxious stimuli. The second class comprises receptors with a low excitability threshold, able to accumulate the intensity of stimulation of harmless stimuli, which, after exceeding a certain critical value, cause sensitization of the receptor by even a weak stimulus. Low-threshold receptors dominate in the large intestine, stomach, bile ducts, and bladder, in contrast to high-threshold receptors prevailing in the lungs, heart, kidneys, and ureters [45]. The presence of silent nociceptive receptors was determined and may constitute even half of the nociceptors in the large intestine and bladder; however, their role is still under investigation [46].

In addition to the abovementioned mechanical stimuli, such as wall distension or organ contraction, the factors triggering visceral pain also include elevated body temperature, ischemia, hypoxia, or inflammation. Rhythmic stimulation of the receptors makes them more sensitive than extero-receptors. Receptor reactions may be possible from low-intensity stimuli that are not harmful [46]. This can happen due to tissue mediators of inflammation (called by Ganong an “inflammatory cocktail”), such as 5-HT, BK, SP, and PGE_2_, which, through their receptors, activate a kinase cascade leading to changes in permeability within Na/K/Ca channels in the cell membrane of the neuron ending, thereby lowering the nociceptor excitability threshold [47]. An important discovery was the isolation of the vanillin receptor (VR1), which is activated not only by temperature changes, but also by hydrogen ions and capsaicin, leading to changes in body temperature that can cause a painful sensation [44,48,49]. Mechanisms of receptor activation by mechanical stimuli are not yet fully understood. Recent studies have demonstrated the existence of purine receptors (P2X_3_), stimulated by ATP released from enterocytes during distension of the organ wall [48].

Regardless of the stimulation of receptors, impulses in afferent fibers (Aδ and C) of the sympathetic (from the thoracic and proximal sacral segment) or parasympathetic (head, neck, remaining sacral segment) system are triggered [41,50]. The bodies of these neurons are in the spinal ganglia within the dorsal roots of the spinal cord. Next, they penetrate together with somatic afferent fibers the gray matter of the spinal dorsal horns. The proximity of these fibers causes visceral pain termed referred (reflective) pain that is transferred to another somatic structure, e.g., heart pain is referred to the left arm, or irritation of the middle part of the diaphragm is felt in the upper part of the shoulder [44]. The existence of viscero-visceral convergences in the spinal cord that affect sensation primarily in the genitourinary system has also been determined [38]. Similarly, there is evidence of viscero-visceral inhibitory reflexes. For example, distension of the duodenal wall and the colon in a sheep inhibits the reticulum and the rumen motility, i.e., the reticulo-ruminal cycles. This reflex was used in our own research to study visceral/intestinal pain.

At the spinal cord level, central sensitization occurs, which is re-sensitization of centripetal neurons to impulses from peripheral neurons. This sensitization happens after stimulation of mGluR N-methyl-D-aspartate (NMDA) receptors with glutamic acid and NK-1 receptors (neurokinins) activated by SP. Inflammation or damage of internal organs enhances the release of these neuro-mediators. Moreover, NMDA receptors have been shown to have magnesium channels, which, under conditions of increased excitation, open, resulting in increased depolarization and activating the NMDA receptor. These receptors mutually stimulate NK-1 receptors and secretion of NO, another mediator of hypersensitivity [34]. Of course, if only stimulatory mechanisms existed in this area, serious homeostasis disorders, damage to the body, shock, and death could occur [50]. Therefore, organisms have developed inhibitory mechanisms, involving γ-aminobutyric acid (GABA), EOPs, and their receptors which are discussed below.

From the spinal cord, the projection travels along ascending pathways of the brain, through spinothalamic tracts (anterior and lateral), the spino-reticular tract, and dorsal columns of the spinal cord (Figure 2). Through dorsal columns, stimuli migrate from the large intestine, pancreas, and duodenum. The intersection of these columns reduces the rectal sensitivity to irritation by up to 80%, while damage to the spinothalamic tracts attenuates them by only 20%, proving the superiority of the dorsal columns over the spinothalamic tracts [48]. Spinothalamic tracts (especially the medial one) branch out to the reticular formation (polysynaptic nonspecific pathways), which explains additional autonomic symptoms associated with pain. Blocking nonspecific pathways by anesthesia eliminates pain [51].

From the thalamus and brainstem, the impulses are transmitted to the limbic system, where emotional evaluation of the pain occurs, and to the sensory cortex, where the pain is localized and perceived [48]. At all three levels of pain conduction (central, spinal, and peripheral), a system for modulation and inhibition of impulses is present. In this system, EOPs play a significant role [1]. The endorphin class includes various types of substances: β-endorphins, dynorphins, and ENKs, as well as casomorphins (milk endorphins) and dermorphins (skin endorphins). ENKs play a superior role in modulating pain sensation. Two types of ENK are known: methionine (Met-ENK) and leucine (Leu-ENK). Both substances are pentapeptides and are products of enzymatic cleavage of the proENK precursor protein (pro-ENK), resulting in the formation of six Met-ENK molecules and one Leu-ENK molecule [52].

The presence of opioid systems has been determined in various brain structures: limbic system, amygdala, septum, striatum, preoptic area, hypothalamus, and brainstem. Three types of receptors specific for EOPs have been distinguished: μ (μ_1_, μ_2_), δ (δ_1_, δ_2_), and κ (κ_1_, κ_2_, κ_3_) [40,41]. High receptor density was found in the central and peripheral nervous system. These receptors are also located in the digestive tract (mainly in intramural ganglia), uterus, spermatic cord, heart, lungs, liver, pancreas, kidneys, and adrenals [42]. ENKs have a high degree of affinity for δ-type opioid receptors and, to a much lesser degree, for μ-type receptors [49].

Opioid receptors are made up of seven transmembrane domains. Part of the N-chain is directed outside the cell, while the C-terminus is directed inward. The molecular mechanism of action of opioid receptors is associated with G_i_ protein. ENK, by binding to a specific receptor, inhibits adenylate cyclase activity, thereby suppressing cAMP synthesis and the cascade of kinases leading to hyperpolarization of the cell membrane. There are also reports about direct (through the inositol cascade) and indirect (inhibition of GABAergic neurons) stimulating the effect of μ and δ receptors. The δ receptors are distributed mainly in the neocortex, caudate nucleus, globus pallidus, septal nuclei, olfactory bulb, amygdala, and pontine nuclei, and they exert their actions via G proteins [52].

Pain modulation occurs at three levels of sensory conduction and is under constant control of the descending systems. Positron emission tomography (PET) methods have proven the existence of two superior structures in the brain responsible for neuromodulation of pain sensation, the periaqueductal gray (PAG) and the rostral ventromedial medulla (RVM) [1]. Both structures receive information from the spinal cord and limbic system. In addition to sending direct ENK-ergic fibers to the spinal cord, neurons of the PAG activate the serotonergic nuclei raphe magnus which, via descending pathways, inhibits the release of SP from the ends of first-order sensory neurons and blocks the transmission of pain information in the first synapse (Figure 2). Moreover, serotonin reaching the substantia gelatinosa of the spinal cord stimulates ENKergic neurons to secrete ENKs that also inhibit SP secretion [52].

It should be emphasized that the entire system of descending neurons is also under the control of ENKs. Large clusters of ENK receptors exist in the PAG and the amygdala (intricately connected to PAG), which regulate the conduction of sensory impulses in the spinal cord [49,50]. The descending NE-ergic system is not subject to this control. The neurons of this system send fibers to the spinal cord with endings secreting NE, which stimulates ENK neurons. Postsynaptic opioid receptors are located in dorsal horns of the spinal cord, primarily on the membrane of interneurons and spinothalamic neurons. These receptors regulate postsynaptic release of SP, CGRP, and CCK [50]. Despite these regulation systems, the nociceptive impulse that moves toward the higher levels of CNS is already inhibited in the first synapse and, thus, the nociception is suppressed. This system was termed “pain gating” and inhibition of the first synapse or “first gate”. The second gate is in the nuclei of the posterior part of the thalamus (nucleus raphe magnus), where an ENK interneuron is situated between the second and third neuron, modulating the signal flow at this level both pre and post synapsis [48].

## 12. Non-Opioid Analgesics

### 12.1. Purpose of the Study

**Figure 5:**
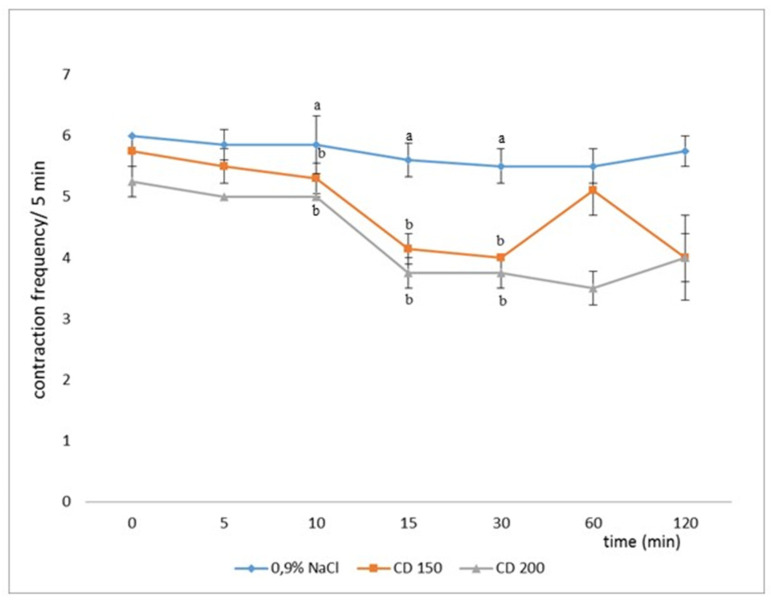
Effect of different degrees of 5 min colonic wall distension with 150 and/or 200 mL of water (CD150 and CD200) on the number of reticulo-rumen contractions before, during, and after 120 min CD in comparison to control (10 mL of 0.9% NaCl; *n* = 6 ± standard error of the mean (SEM)); a—statistically significant in relation to CD150, b—statistically significant in relation to control [18].

The aim of the study was a comparative analysis of the results obtained in our laboratory on the experimental search for alternative analgesics in sheep visceral/intestinal pain on a model of intestinal colic caused by mechanical distension of the duodenal wall (Figure 5). The results acquired in this way were compared with those obtained in a less invasive method—the descending colonic wall distension model in a sheep. There are surprisingly few reports on the rational use of opioids in pain mitigation in sheep (29 reviewed manuscripts in 1995–2018 [13], in which many—including the author’s five reviewed manuscripts—were omitted) and even fewer of those regarding testing the suitability of non-opioid compounds for analgesic purposes, which our laboratory has been doing for many years.

Paradoxical reactions, especially behavioral responses after opioid use in ruminants, have led to the search for non-opioid agents with potential analgesic effects. First, CCK antagonist substances were studied for their inhibition of EOP’s analgesic effects. Subsequently, VGCCIs from various chemical groups were tested, because nifedipine is known to increase morphine analgesia and prevent addiction. We compared the effects of nifedipine with two L-type L2 calcium channel antagonists, diltiazem (benzodiazepine) and verapamil (phenylethylalkylamine). In the next stage, we examined the analgesic effects of L-AP_3_ and DL-AP_3_, antagonists of mGluR, because mGluR5 antagonists have analgesic effects with experimentally induced, neuropathic pain in rodents [17,53,54].

### 12.2. Methods and Results

**Figure 6:**
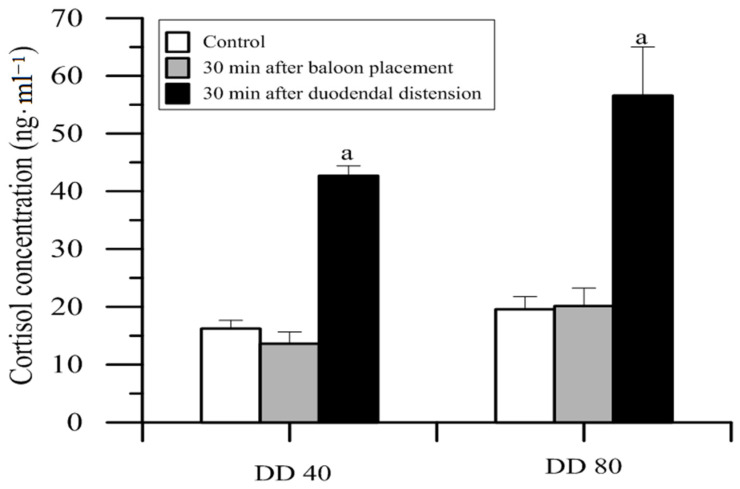
Effect of duodenal wall distension on cortisol concentration in sheep plasma in comparison to double control (*n* = 6 ± SEM) [18]. DD40—distension by 40 mL of water; DD80—distension by 80 mL of water, a – statistically significant in relation to the previous groups

**Figure 7:**
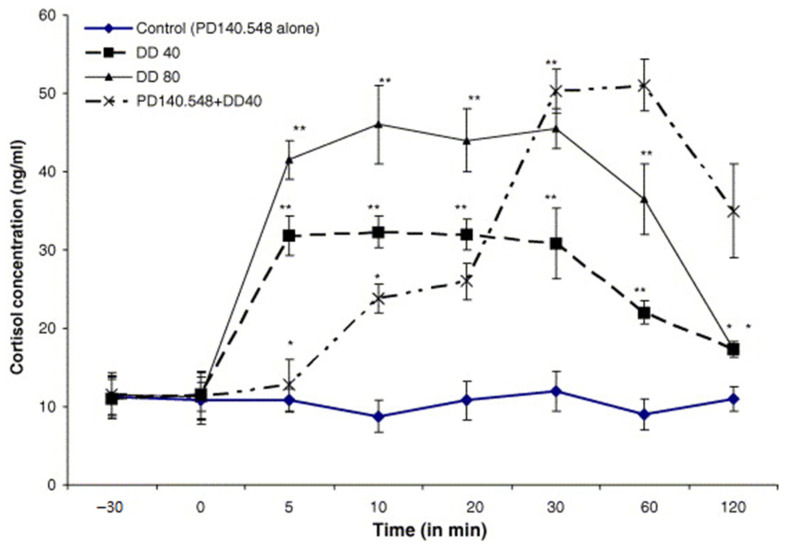
Comparative analysis of PD140.548 (intracerebroventricular (ICV) infusion) before and after duodenal distension (DD40 and DD80) on changes in plasma cortisol concentrations in comparison to controls (100 µL of 0.9% NaCl), (*n* = 6 ± SEM). Points that are significantly different from control are marked with asterisks [50]; * *p* < 0.05 and ** *p* < 0.01.

**Figure 8:**
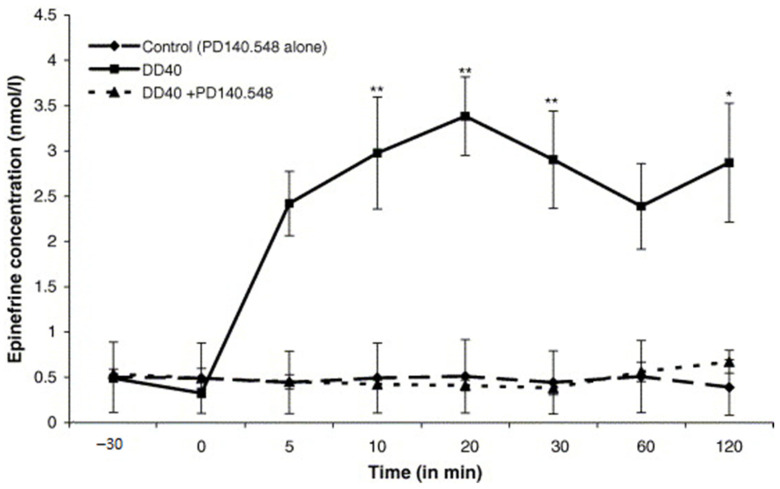
Comparative analysis of PD140.548 (ICV infusion) on changes in plasma epinephrine concentrations before and after 5 min of DD40 episode in comparison to controls (100 µL of 0.9% NaCl, *n* = 6 ± SEM). Points that are significantly different from control are marked with asterisks [50]; * *p* < 0.05 and ** *p* < 0.01.

**Figure 9:**
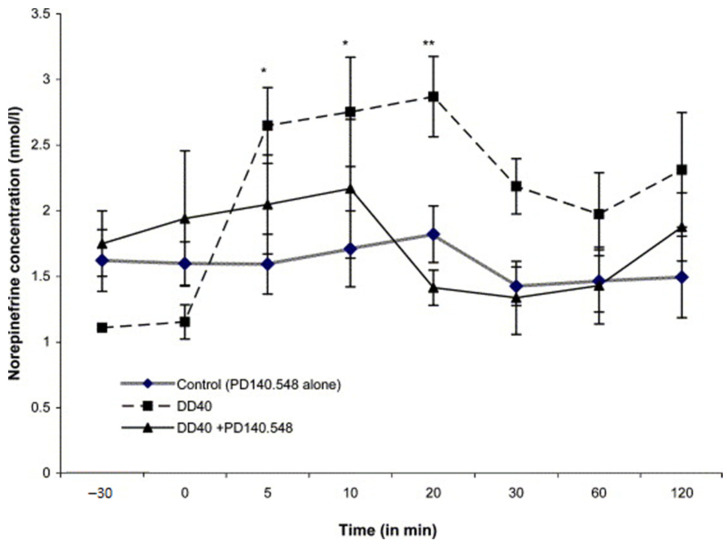
Comparative analysis of PD140.548 (ICV infusion) on changes in plasma norepinephrine concentrations before and after 5 min of DD40 episode in comparison to controls (100 µL of 0.9% NaCl, *n* = 6 ± SEM). Points that are significantly different from control are marked with askterisks [50]; * *p* < 0.05 and ** *p* < 0.01.

**Figure 10:**
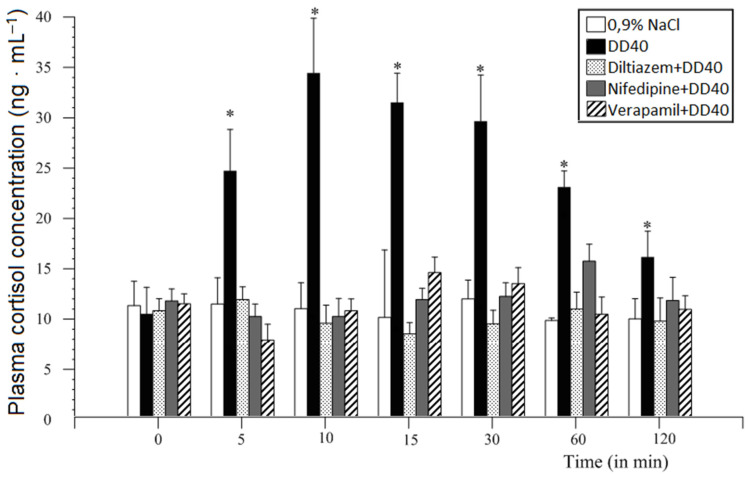
Comparative analysis of 10 min (ICV infusion) premedication influence of diltiazem, nifedipine, and/or verapamil (in doses 1.0 and/or 2.0 mg/animal) and DD40 on plasma cortisol concentration in sheep in comparison to DD40 value at *p* ≤ 0.001–0.05 (mean ± SEM, *n* = 6). Significant differences are marked with asterisks [34,50].

**Figure 11:**
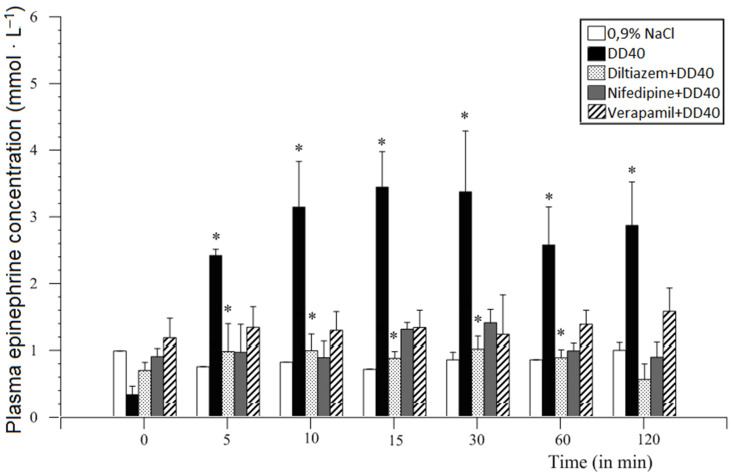
Comparative analysis of duodenal distension and premedication with different doses of diltiazem, nifedipine, and verapamil doses (1.0 or 2.0 mg/animal) on plasma epinephrine concentration in comparison with DD40 (mean ± SEM, *n* = 6, *p* ≤ 0.001–0.05). Mean values of results obtained from the same blood collection (time point). Significant differences are marked with asterisks [34,50].

**Figure 12:**
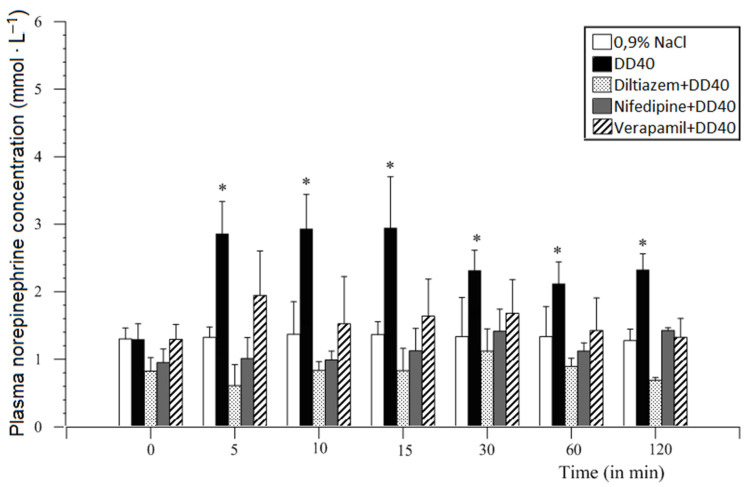
Comparative analysis of duodenal distension and premedication with different doses of diltiazem, nifedipine, and verapamil (1.0 or 2.0 mg/animal) on plasma norepinephrine concentration in comparison with DD40 (mean ± SEM, *n* = 6, *p* ≤ 0.001–0.05). Mean values of results obtained from the same blood collection (time point). Significant differences are marked with asterisks [34,50].

**Figure 13:**
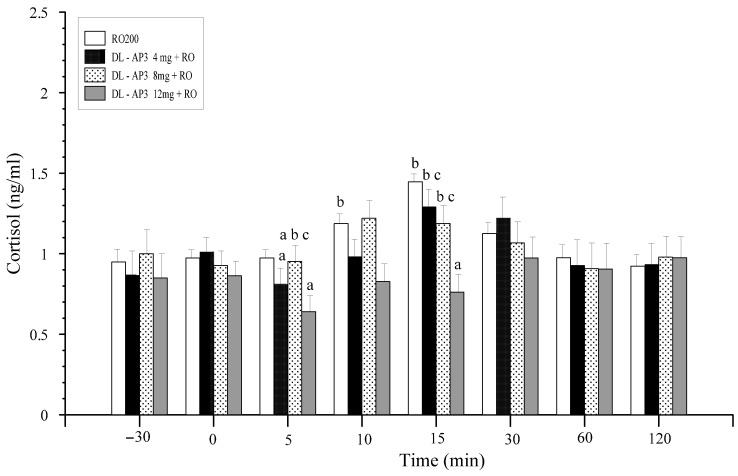
Summed from three doses, mean changes in plasma cortisol levels (ng·mL^−1^) in the test groups of animals. Control group (200 μL of 0.9% NaCl), RO200, and after ICV infusion 10 min premedication with three different doses of L-AP_3_ (0.2, 0.4, and/or 0.8 mg in toto) and DL-AP3 (2, 4, and/or 8 mg in toto) in the RO200 test within 120 min of completing the RO procedure (mean ± SD, *n* = 6) [34]. Key: a—significant differences (*p* ≤ 0.05) compared with the value of 0.9% NaCl; b—significant differences (*p* ≤ 0.05) compared with the value of the RO200 group; c—significant differences (*p* ≤ 0.05) between the L-AP_3_ and DL-AP_3_ groups.

**Figure 14:**
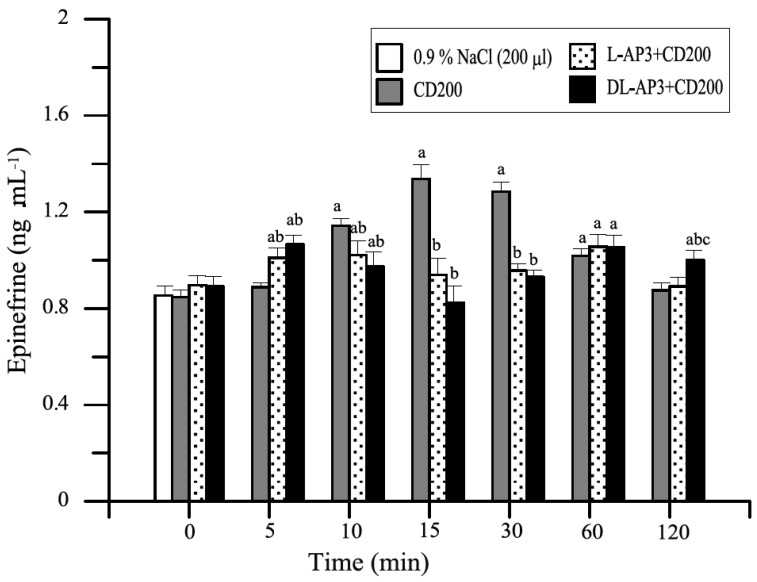
Summed from three doses, mean changes in plasma epinephrine levels (ng·mL^−1^) in the test groups of animals. Control group (200 μL of 0.9% NaCl), RO200, and after 10 min (ICV infusion) premedication with three different doses of L-AP_3_ (0.2, 0.4, and/or 0.8 mg in toto) and DL-AP_3_ (2, 4 and/or 8 mg in toto) in the RO200 test within 120 min of completing the RO procedure (mean ± SD, *n* = 6) [34]. Key: a—significant differences (*p* ≤ 0.05) compared with the value of 0.9% NaCl; b—significant differences (*p* ≤ 0.05) compared with the value of the RO200 group; c—significant differences (*p* ≤ 0.05) between the L-AP_3_ and DL-AP_3_ groups.

**Figure 15:**
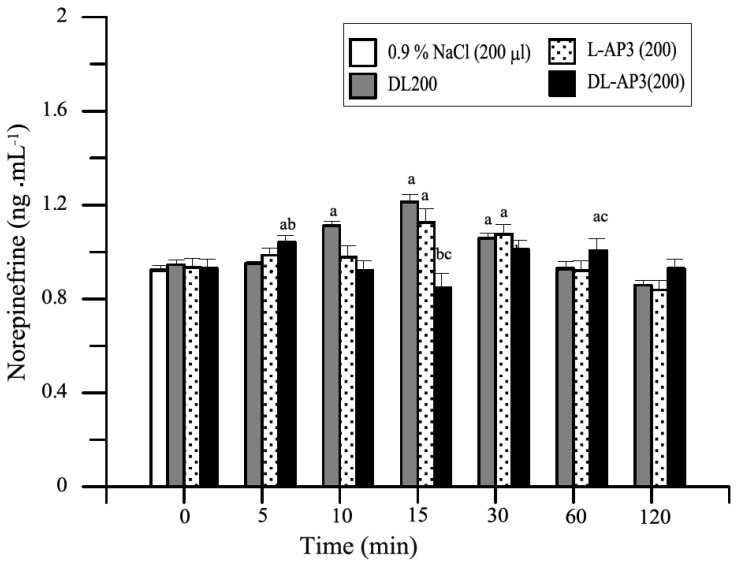
Summed from three doses, mean changes in plasma norepinephrine levels (ng·mL^−1^) in the test groups of animals. Control group (200 μL of 0.9% NaCl), RO200, and after 10 min (ICV infusion) premedication with three different doses of L-AP_3_ (0.2, 0.4, and/or 0.8 mg in toto) and DL-AP_3_ (2, 4, and/or 8 mg in toto) in the RO200 test within 120 min of completing the RO procedure (mean ± SD, *n* = 6) [34]. Key: a—significant differences (*p* ≤ 0.05) compared with the value of 0.9% NaCl; b—significant differences (*p* ≤ 0.05) compared with the value of the RO200 group; c—significant differences (*p* ≤ 0.05) between the L-AP_3_ and DL-AP_3_ group.

The aim of this study was to determine the influence of 5 min mechanically induced duodenal distension (DD), proglumide, and PD 140.548 *N*-methyl-d-glucamine (a specific peptide antagonist of a CCK_1_ receptor) premedication on mechano-graphical, reticulo-ruminal activity, animal general behavior (according to method earlier described [13,50]), CA, and the blood plasma cortisol levels, as well as the clinical symptoms of visceral pain induced by DD in sheep (30 males, 3–4 years of age) (Table 4 and Table 5, Figure 6, Figure 7, Figure 8, Figure 9, Figure 10, Figure 11, Figure 12, Figure 13, Figure 14 and Figure 15). After 24 h fasting, six Polish merino sheep were preanesthetized by intramuscular (i.m.) injection of ketamine (20 mg·kg^−1^ body weight (b.w.)) and anesthetized with intravenous (i.v.) infusion of pentobarbital (20 mg·kg^−1^ b.w.), and a permanent stainless-steel cannula (gate cannula) was inserted inside the lateral cerebral ventricle (controlled by cerebrospinal fluid efflux), 10 mm above the bregma and 5 mm laterally from the midline suture using stereotaxic method. Under the same general anesthesia and analgesia, a T-shaped silicon cannula was inserted into the duodenum (12 cm from pylorus) and a second one was inserted into the dorsal sac of the rumen. For 7 consecutive days after surgery each animal was treated i.m. with procaine penicillin (300,000 I.U.·kg^−1^ b.w.), dihydrostreptomycin (DHS, 10 µg·kg^−1^ b.w.), prednisolone acetate (1.2 mg·kg^−1^ b.w.), and a second i.m. injection of ketamine (20 mg·kg^−1^ b.w.). The influence of proglumide or PD 140.548 *N*-methyl-d-glucamine on the unfavorable effects of DD using a 10 cm long balloon filled with 40 and 80 mL (DD40 and DD80) and 150 or 200 mL of water (CD) at animal body temperature was investigated. Five minutes of DD40, DD80, CD150, and CD200 caused an immediate and complete inhibition of the reticulo-ruminal frequency, a significant increase in plasma CA and cortisol levels, and an increase in the heart rate, hyperventilation, and other symptoms of pain, which were proportional to the degree of intestinal distension (*p* ≤ 0.05 according to SIGMA Stat 2.03 pprogram). Intracerebroventricular (ICV) administration of PD 140.548 alone at a dose of 0.25, 0.5, 1, or 2 mg in toto did not significantly change the reticulo-ruminal motility, CA, and cortisol concentrations; however, 10 min after the ICV infusion (or 10 min before DD) at a dose of 1 and 2 mg in toto, it completely blocked the increase in blood plasma cortisol, epinephrine (E), norepinephrine (NE), and dopamine (DA) concentrations for 20 min. Reticulo-ruminal atony provoked by DD or CD was also prevented (*p* ≤ 0.05). Therefore, PD 140.548 *N*-methyl-d-glucamine and proglumide, an antagonist of the central CCK_1_ receptor, can be an effective analgesic agent in duodenal pain. This action is due to the inhibition of peripheral CCK_1_ type receptor in the central descending nerve pathway, facilitating pain transmission in sheep perhaps in the hypothalamic–pituitary–adrenal (HPA) axis [34]. After the study, the animals were euthanized. All experimental procedures were approved by the Local Bioethics Committee at the Jagiellonian University in Cracow, Poland (approval No 75/2007) and were conducted at the Department of Animal Physiology and Endocrinology of Agricultural University in Cracow (Poland).

Data were statistically analyzed by two-way ANOVA followed by Duncan’s multiple range test. Log transformation was performed as needed to maintain homogeneity of variance and normality. Differences of values were considered significant at *p* < 0.05. Calculations were performed using a Sigma Stat 2.03 program (SPSS Science Software GmbH, Germany). Results are presented as mean ± SE [34].

### 12.3. Inhibitors of Cholecystokinin (CCK)

CCK released in the CNS inhibits the analgesic action of exogenous opioids and may antagonize analgesia resulting from the activation of an endogenous pain inhibitory system. The aim of this study was to analyze the central action of PD 140.548 *N*-methyl-d-glucamine and proglumide, a peptide antagonist of a specific peripheral type CCK receptor, on sheep behavior (Table 4), plasma catecholamines (CA), and cortisol concentration (Figure 6, Figure 7, Figure 8 and Figure 9), as well as clinical symptoms of visceral pain induced by duodenal (DD) or descending colon distension (CD) in different doses (Figure 5) [34].

### 12.4. Voltage-Dependent Calcium Channel Inhibitors

In the experiments, the same biochemical and behavioral parameters were determined as for CCK1 receptor antagonists. Different doses of diltiazem, nifedipine, and/or verapamil (L-VGCC antagonists from three different chemical groups) were used. The obtained results are presented in tables and charts.

ICV infusion of VGCCIs in 10 min premedication prevented nocifensive signs of behavior and clinical symptoms, as well as increased plasma cortisol and catecholamine concentration in periphery and perhaps in CNS structures. The molecular mechanisms of these processes are the result of the L-type voltage-gated calcium channel inhibitor blockage of specific Ca^2+^ receptors by the drugs tested. Calcium channel receptor blockage by VGCC inhibitors attenuates visceral pain by inhibiting nocifensive neurohormone/neurotransmitter release in the CNS and in peripheral nervous system, due to Ca^2+^ ions’ inability to bind to their specific receptor for depolarizing the presynaptic neuronal membrane and promoting the release of nocifensive substances [34,46].

### 12.5. Inhibitors of Metabotropic Glutaminergic Receptors (mGluR_1_)

Glutamic acid and its mGluR receptors play important roles in the mechanisms of induction and transmission of nociceptive stimuli. The substances L-AP_3_ and DL-AP_3_ were examined to determine their effect on changes caused by distension with 150 and/or 200 mL of water (CD150 or CD200) of the descending colonic wall before and after the application of CD. The results are presented on Figure 13, Figure 14 and Figure 15 [19,50].

## 13. Discussion

In this study, the nocifensive factor, acting one time for 5 min, triggered a generalized emotional response in the sheep’s body as a reaction to a stress factor. Visceral/intestinal pain caused by distension of the descending colonic wall with a balloon containing 150 or 200 mL of water at the animal’s body temperature after 5 min caused an increase in plasma cortisol concentration by about 75%, indicating intense stress and stimulation of the HPA axis. Repetition of pain provocation in the same animals at weekly intervals reduced the intensity of the stress response, increasing hormone concentration on average by only 35% [34].

It should be noted that, in a comparable experiment, a 5 min distension of the duodenal wall with a rubber balloon containing 40 and/or 80 mL of water at the animal’s body temperature increased the plasma cortisol concentration by about 420% [48]. These differences appear to be a result of the anatomical and neurophysiological properties of the individual parts of the sheep’s digestive tract. The colon constantly contains fecal masses; thus, both the mucosa and its nociceptors are to some extent adapted to this condition. Results indicate that both forms of AP_3_, a non-specific antagonist of metabotropic glutaminergic receptor, can be recommended for sheep as both an analgesic and an antistress agent because the AP_3_ racemate was also effective in preventing autonomous symptoms, including the viscero-visceral inhibitory reflex to visceral pain caused by distension of the descending colonic wall in sheep [19,34]. The observed decrease in plasma cortisol concentration by AP_3_ suggests inhibition of central mGluR1 receptors in structures controlling sensation and conduction of pain (spinal dorsal horn, amygdala, reticular formation, medulla oblongata, and PAG) and emotions, especially in central structures regulating emotional states and motivations (amygdala, hippocampus, hypothalamus, and prefrontal cortex). Blocking receptors of the amygdala and paraventricular nucleus (PVN) and subthalamic nucleus (STN) of the hypothalamus prevents the depolarization of corticotropin-releasing factor (CRF)-releasing neurons and interrupts the cascade of phenomena related to the activation of the HPA axis and the release of glucocorticosteroids (CRF→ACTH→cortisol), as well as the stimulation of the sympathoadrenal system (SAS) with subsequent release of catecholamines from the adrenal medulla [18]. The reaction to such stress factors is abolished and/or suppressed; thus, the experimental procedure can be used to effectively improve animal welfare.

## 14. Conclusions

In conclusion, 5 min distension of the descending colonic wall with a balloon filled with 150 and/or 200 mL of water is a proper model for causing psycho-physical stress, manifested by increased plasma cortisol, catecholamines, and vegetative behavioral symptoms.

## Figures and Tables

**Table 1:** Distribution of opioid receptors (ORs) in organs.

Receptor	Subtypes	Location	Function	G Protein Subunit
delta (δ) DOR OP_1_^(I)^	δ_1_, δ_2_	brain ○ pontine nuclei ○ amygdala ○ olfactory bulbs ○ deep cortex peripheral sensory neurons	analgesia antidepressant effects convulsant effects physical dependence may modulate μ-opioid receptor-mediated respiratory depression	G_i_
kappa (κ) KOR OP_2_^(I)^	κ_1_, κ_2_, κ_3_	brain ○ hypothalamus ○ periaqueductal gray ○ claustrum spinal cord ○ substantia gelatinosa peripheral sensory neurons	analgesia anticonvulsant effects depression dissociative/hallucinogenic effects diuresis miosis neuroprotection sedation stress	G_i_
mu (μ) MOR OP_3_^(I)^	μ_1_, μ_2_, μ_3_	brain ○ cortex (laminae III and IV) ○ thalamus ○ striosomes ○ periaqueductal gray ○ rostral ventromedial medulla spinal cord ○ substantia gelatinosa peripheral sensory neurons intestinal tract	**μ_1_**: analgesia physical dependence **μ_2_**: respiratory depression miosis euphoria reduced GI motility physical dependence **μ_3_**: possible vasodilation	G_i_
Nociceptin receptor NOR OP_4_^(I)^	ORL_1_	brain ○ cortex ○ amygdala ○ hippocampus ○ septal nuclei ○ habenula ○ hypothalamus spinal cord	anxiety depression appetite development of tolerance to μ-opioid agonists	
zeta (ζ) ZOR		heart liver skeletal muscle kidney brain pancreas fetal tissue ○ liver ○ kidney	tissue growth ○ embryonic development ○ regulation of cancer cell proliferation	

(I)—name based on order of discovery.

**Table 2:** Functional activity associated with various types of opioid receptors (according to [1]).

	Receptor Subtype	µ	δ	κ
Effect				
Analgesia				
Supraspinal		+++	−	−
Spinal		++	++	+
Peripheral		++	−	++
Inhibition of respiration		+++	++	−
Miosis		++	−	+
Inhibition of gastrointestinal motility		++	++	+
Euphoria		+++	−	−
Dysphoria		−	−	+++

+++ very high activity, ++ high activity, + poor activity, − no activity. Currently, some authors also distinguish subclasses of these opioid receptor types (µ_1_, µ_2_, δ_1_, δ_2_, κ_1_, κ_2_, κ_3_) [9].

**Table 3:** Actual and previous terminology of opioid receptors according to *The International Union of Basic and Clinical Pharmacology* (IUPHAR), their ligands, and the genes encoding them (according to [29]).

Currently Used Terminology According to the IUPHAR Committee on Receptor Nomenclature and Drug Classification	Previous Terminology	Main Endogenous Agonist	Genes
m-receptor (m *receptor*) (MOP, *μ-opioid peptide*) receptor	MOR-1, MOR (*mu-opioid receptor*) OP3	[β-endorphin] [Met]enkefalin [Leu]enkefalin Endomorphin 1 and 2	*OPRM-1* (Hs), *Oprm1* (Mm), *Oprm1* (Rn) ?
d-receptor (d *receptor*), (DOP, *μ-opioid peptide*) receptor	DOR-1, DOR (*delta opioid receptor*) OP1	[Met]enkephalin [Leu]enkefalin)d β-endorphin	*OPRD1* (Hs), *Oprd1* (Mm), *Oprd1* (Rn)
k-receptor (k *receptor*) (KOP, k-*opioid peptide*) receptor	KOR-1, KOR (*kappa-opioid receptor*) OP2	Big dynorphin Dynorphin A α-neoendorphin	*OPRK1* (Hs), *Oprk1* (Mm), *Oprkd1* (Rn)

?—not yet identified.

**Table 4:** The effect of duodenal distension by 40 mL of water (DD40) on the ruminal motility (inhibition in %/5 min in comparison to control values) and behavioral symptoms (number/5 min) in sheep before and after voltage-gated calcium channel inhibitor (diltiazem—1, nifedipine—2 and verapamil—3) pretreatment at a dose of 1 or 2 mg in toto (i.e., 25 or 50 µg·kg^−1^ body weight (b.w.); *n* = 6).

Accompanying Symptoms	0–5				5–10				10–15				25–30				55–60				120 min			
	DD	1	2	3	DD	1	2	3	DD	1	2	3	DD	1	2	3	DD	1	2	3	DD	1	2	3
Inhibition of ruminal activity	4+	−	+	−	4+	−	±	−	3+	2	+	+	−	−	−	−	−	−	−	−	−	−	−	−
Looking around	3+	±	±	±	2+	±	±	±	+	+	+	+	−	−	−	+	−	−	−	−	−	−	−	−
Defecation	3+	−	−	−	+	−	±	−	−	±	−	±	−	−	±	+	−	−	−	−	−	−	−	−
Head movements	3+	−	−	−	2+	±	±	±	−	−	−	−	−	−	±	+	−	−	−	−	−	−	±	−
Stretching	2+	−	−	−	+	−	−	−	−	−	+	±	−	−	−	−	−	−	−	−	−	−	−	−
Grinding	2+	±	±	±	−	+	+	+	−	±	±	±	−	−	−	−	−	−	−	−	−	−	−	−
Lying down	2+	−	−	−	+	−	−	−	−	±	±	−	−	−	−	−	−	−	−	−	−	−	−	−
Bleating	+	−	−	−	+	−	−	−	−	−	−	−	−	+	+	+	+	−	−	−	−	−	−	−
Tachycardia	4+	±	3+	±	4+	−	3+	±	4+	−	3+	±	3+	−	±	±	3+	−	±	±	3+	−	−	−
Hyperventilation	4+	±	−	±	3+	−	−	±	4+	−	−	±	3+	−	−	−	3+	−	−	−	3+	−	−	−

4+ very strong, 3+ strong, 2+ quite strong, +/− from time to time, − no effect [34,50].

**Table 5:** Frequency of ruminal contraction of the five groups (control, DD40, diltiazem + DD40, nifedipine + DD40, verapamil + DD40) during the experiments [34].

Drugs	Control	5 min	10 min	15 min	20 min	25 min	30 min
0.9% NaCl	6.45 ± 0.75	6.12 ± 0.28	6.00 ± 0.25	6.80 ± 0.33	6.10 ± 0.60	6.35 ± 0.15	6.11 ± 0.43
DD40	6.15 ± 0.54	1.09 ± 0.33 *	1.78 ± 0.49 *	1.35 ± 0.52 *	2.20 ± 0.31 *	0.61 ± 0.12 *	4.91 ± 0.75
Diltiazem + DD40	5.00 ± 0.61 *	4.00 ± 0.38 *	3.80 ± 0.60 *	4.23 ± 0.36 *	5.14 ± 0.42	4.82 ± 0.64	6.12 ± 0.40
Nifedipine + DD40	5.82 ± 0.45	1.89 ± 0.81 *	5.54 ± 0.23	4.88 ± 0.62	5.12 ± 0.74	6.11 ± 1.11	5.59 ± 1.22
Verapamil + DD40	6.12 ± 0.89	5.33 ± 0.51	5.75 ± 0.11	5.05 ± 0.80	4.97 ± 0.65	5.55 ± 1.02	5.85 ± 0.61

Values are the mean ± SEM of six sheep, and indicate significant differences corresponding to the control group (mean ± SEM, *n* = 6, * *p* ≤ 0.001–0.05). Significantly different results are marked with asterisks.
